# Postmortal epithelial changes of donor corneas impair applicability of a refractive ultraviolet femtosecond laser

**DOI:** 10.1038/s41598-025-89867-4

**Published:** 2025-02-12

**Authors:** Christian M. Hammer, Marius Topka, Yao Zhang, Thilo Hotfiel, Friedrich Paulsen, Alexey Larionov, Johannes Lörner

**Affiliations:** 1 Anatomy Unit, Section of Medicine, Faculty of Science and Medicine, University of Fribourg, Route Albert-Gockel 1, Fribourg, 1700 Switzerland; 2 Institute of Functional and Clinical Anatomy, Friedrich-Alexander-Universität Erlangen-Nürnberg, Universitätsstraße 19, 91054 Erlangen, Bavaria Germany; 3 WaveLight GmbH, Am Wolfsmantel 5, 91058 Erlangen, Bavaria Germany; 4 Department of Orthopedic and Trauma Surgery, Friedrich-Alexander-Universität Erlangen-Nürnberg, Krankenhausstraße 12, 91054 Erlangen, Bavaria Germany

**Keywords:** LASIK, SMILE, KLEx, Refractive surgery, Femtosecond laser, Flap creation, Lasers, LEDs and light sources, Translational research

## Abstract

This study evaluates the corneal applicability of a refractive ultraviolet femtosecond laser in postmortal human donor eyes and ex vivo porcine eyes. Refractive lenticule extraction and flap creation were attempted in 10 human donor eyes and 80 ex vivo porcine eyes with and without abrasion of the corneal epithelium. The postmortem interval ranged from 6 to 35 h in the human samples and was set to 4, 24, and 48 h for the porcine specimens. Nine human eyes and 60 porcine eyes were treated with an ultraviolet femtosecond laser. The rest was treated with an infrared laser. Optical coherence tomography and scanning electron microscopy were used to demonstrate success or failure of the procedures. Ultraviolet laser-assisted refractive surgery attempts without prior abrasion of the corneal epithelium were only successful at 6 h p.m. in the human eyes and at 4 and 24 h in the porcine eyes. Upon epithelial abrasion, refractive surgery was always successful with the ultraviolet laser. The infrared laser always performed successfully with and without prior epithelial abrasion. Thus, postmortal changes in the corneal epithelium impair the ability of refractive ultraviolet femtosecond lasers to create stromal cuts. This progresses with time but does not affect infrared femtosecond lasers.

## Introduction

Femtosecond lasers have become indispensable tools for refractive corneal surgery^1^. In the context of laser-assisted in situ keratomileusis (LASIK) they have replaced the mechanical microkeratome for the purpose of ultraprecise stromal flap creation (which is then followed by stromal photoablation by an excimer laser)^2^. This way, the safety of the procedure and the predictability of its outcome have improved substantially^3–5^. Another prime example is keratorefractive lenticule extraction (KLEx) – a set of procedures that rely completely on femtosecond lasers^6^. Here, the femtosecond laser is used to cut a lens-shaped piece of stroma (refractive lenticule) in the depth of the cornea via photodisruption^7^. This is ensued by manual lenticule extraction either through a corneal incision^8^ or after lifting a stromal flap^9^. The flapless version is also known as small incision lenticule extraction (SMILE)^7,8^. It has the advantage of leaving the anterior stroma largely unscathed, thus minimizing the wound size and the biomechanical destabilization of the cornea^7,10^. The creation of both, flaps and lenticules, is usually performed by laser systems emitting in the near infrared wavelength domain^11^. However, shorter wavelengths have the advantage of allowing for a shorter laser focus, resulting in optical breakdown and disruption of the corneal stroma at considerably lower pulse energies^12^. This, in turn, is likely to decrease collateral tissue damage and increases the highest possible degree of surgical precision^13^. For these reasons, a refractive ultraviolet femtosecond laser system emitting at 347 nm has been developed by Alcon-WaveLight (WaveLight GmbH, Erlangen, Germany) for LASIK and KLEx^14^. In its preclinical testing phase, the laser was shown to create stromal cuts with only a fraction of the pulse energy necessary with its infrared counterparts^15,16^. It was also demonstrated to pose no threat in terms of collateral corneal damage, detrimental wound healing, cataract formation, or retinal injury^16–18^. To the contrary, it markedly surpassed its competing infrared laser platforms with regards to the minimization of interface roughness and gas formation^15^. Hence, it was to some degree predictable that this novel ultraviolet femtosecond laser platform successfully passed the preclinical testing phase. Since the underlying experiments had largely been performed in ex vivo porcine eyes, the next logical step would have been to repeat them in human donor eyes. Surprisingly, however, the first preliminary attempts to cut and extract refractive lenticules in the laboratory failed (see below). Only after removal of the corneal epithelium was it possible to create a lamellar cut with the ultraviolet femtosecond laser. As will be described in detail in this study, extremely short postmortem times, similar to those achievable for porcine eyes obtained from the local abattoir (i.e. less than six hours), allowed for stromal laser cuts with the epithelium still in place. Interestingly, none of these problems were encountered using a standard infrared femtosecond laser system. From this set of preliminary data acquired from a limited number of human donor eyes (*n* = 10), the following three hypotheses were generated: (1) Postmortal changes in the corneal epithelium impair the creation of stromal ultraviolet femtosecond laser cuts. (2) These epithelial changes progress with time. (3) The postmortal epithelial changes interfere with ultraviolet, but not with infrared femtosecond lasers. Since human donor eyes are very precious and not available in large quantities, these hypotheses were tested with enucleated, abattoir-acquired pig eyes. The present study provides a detailed report on the above-mentioned preliminary attempts in human eyes that had led to the formulation of the working hypotheses. It also describes the procedure and the outcome of hypothesis testing with porcine eyes.

## Materials and methods

### Study design

#### Human eyes

A total of 10 human eyes was enucleated from 5 body donors (see Table 1) at the Institute of Functional and Clinical Anatomy, Friedrich-Alexander-Universität Erlangen-Nürnberg, Erlangen, Germany. Upon enucleation, the globes were stored in a humid chamber and kept refrigerated (4 °C) until further use. In 8 of those eyes (taken from 4 body donors), refractive lenticule creation was attempted with the novel ultraviolet femtosecond laser (see below). The experimental parameters like postmortem time, intended refractive correction, and epithelial abrasion varied from eye to eye (see Table 1). In case of successful lenticule extraction, a side cut was created and the lenticule cap was lifted like a flap to expose the lenticule bed. The remaining 2 eyes of the 5th body donor were used for flap creation with the ultraviolet laser (left eye) and an infrared femtosecond laser platform (right eye). These flaps were also lifted to expose the corneal stroma. Success or failure of the lasers to create lamellar cuts were verified by anterior segment optical coherence tomography (AS-OCT), scanning electron microscopy (SEM), and by the investigators’ ability to dissect and extract the lenticules or lift the flaps. Generally, the corneal epithelium remained intact at the first attempt. In most cases (see Table 1), the epithelium was abraded with a hockey knife upon cutting failure, before the second attempt. In those cases where lenticule extraction and/or flap lift had been possible, the morphology of the resulting lamellar beds was examined by use of SEM. All human material was harvested and used for research with the body donors’ written and informed consent that they had given during their lifetime. According to applicable local regulations at the Friedrich-Alexander-Universität Erlangen-Nürnberg, Germany and governing German law, an additional approval by the ethics committee was not necessary. All experiments have been performed in accordance with the Declaration of Helsinki.

**Table 1:** Donor and experimental sample parameters.

Donor	Age (y)	Sex	p.m. (h)	Eye	Laser	Cut	Correction	Epithelial abrasion
#1	76	m	15	Left	UV	Lenticule	− 5 D	No
				Right	UV	Lenticule	− 5 D	No
#2	88	m	34	Left	UV	Lenticule	− 5D	**1st att.**: no, **2nd att.**: yes
				Right	UV	Lenticule	− 10 D	Yes
#3	70	m	35	Left	UV	Lenticule	− 5 D	**1st att.**: no, **2nd att.**: yes
				Right	UV	Lenticule	− 8 D	Yes
#4	69	w	**6**	Left	UV	Lenticule	− 5 D	Yes
				Right	UV	Lenticule	− 5 D	**No**
#5	77	w	33	Left	UV	Flap	n.a.	**1st att.**: no, **2nd att.**: yes
				Right	**IR**	Flap	n.a.	No

The attempted myopic correction by lenticule extraction is given in diopters (D). Abrasion: removal of the corneal epithelium with an ophthalmological hockey knife (*att:* attempt). Significant values are in bold.

#### Porcine eyes

**Fig. 1:**
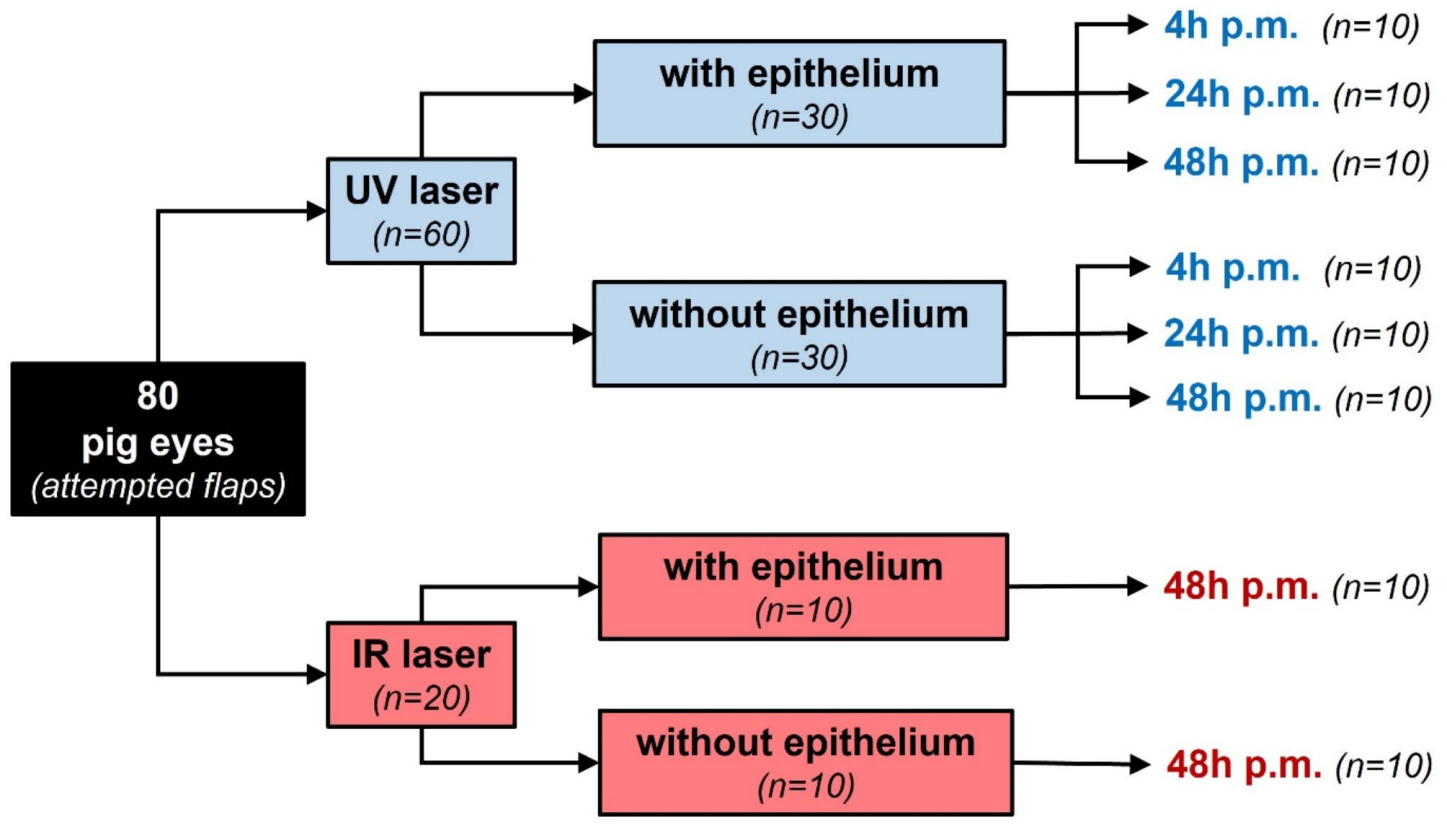
Study design pertaining to hypothesis testing. In 80 porcine eyes, stromal flap creation was attempted with a femtosecond laser. For this, an ultraviolet (*UV*) and an infrared (*IR*) femtosecond laser were used. While the IR laser-related control experiments were conducted after 48 hours (*h*) postmortem (*p.m*.) only, the UV laser was applied after 4, 24, and 48 hours. In every experimental group, half of the samples received laser treatment with the corneal epithelium still in place, and the other half after epithelial abrasion.

Eighty porcine eyes that had been acquired from the local abattoir (Contifleisch GmbH, Erlangen, Germany) were used in the present study. Care was taken that the eyes had been harvested at the slaughterhouse after the kill (exsanguination after electronarcosis), but before scalding of the carcasses^19,20^. Animal treatment was carried out by experienced abattoir staff and was supervised and approved by the local veterinary office (Amt für Veterinärwesen und gesundheitlichen Verbraucherschutz, Abteilung Fleischhygiene, Erlangen, Germany). The pig eyes were kept refrigerated (4 °C) in a humid chamber until further use. In 60 eyes, stromal flap creation was attempted with an ultraviolet femtosecond laser developed by Alcon-WaveLight (WaveLight GmbH, Erlangen, Germany). One third (*n* = 20) was subjected to flap creation, immediately (i.e. after 4 h postmortem). The other two thirds received laser treatment after 24 h (*n* = 20) and 48 h (*n* = 20), respectively. In each of these subgroups, one half of the eyes underwent laser treatment with the corneal epithelium still in place (*n* = 10), in the other half, the epithelium had been abraded with a hockey knife prior to surgery (*n* = 10). Success or failure of flap creation were verified by AS-OCT, SEM, and by the investigators’ ability to dissect and lift the flaps. In those cases where the flap lift had been possible, the morphology of the resulting lamellar beds was examined by use of SEM. The remaining 20 eyes served as a comparative control and were subjected to flap creation with an infrared femtosecond laser at 48 h postmortem. Of these, one half underwent laser treatment with the corneal epithelium still intact (*n* = 10), and the other half after abrasion with a hockey knife (*n* = 10). After that, the eyes in the infrared laser group were treated in the same way as the ones that had been lasered with the ultraviolet system. An overview of the study design pertaining to the porcine eyes is given in Fig. 1.

### Laser parameters

The ultraviolet femtosecond laser platform that was used in the present study had already been described extensively in previous works^14–19^. It was operated with a repetition rate of 1000 kHz. Flap and lenticule creation were attempted with a pulse energy of 80 nJ, a pulse duration of ~ 300 fs, a spot size of 1.7 ± 0.2 μm, and a spot separation of 4 μm x 4 μm (total fluence: 1.0 J/cm²). These are also the normal settings for clinical application. In case of successful flap or lenticule formation, the remaining tissue bridges across the interfaces were severed manually with a blunt spatula. After lenticule extraction through a small extraction canal, a side cut was applied to enable lifting of the lenticule cap. The diameter of the lenticules (optical zone) was set to 6.5 mm. The anterior interfaces of the lenticules as well as the flap interfaces were aimed at a corneal depth of 130 μm. The flaps had a uniform diameter of 9.0 mm. Where possible, the lenticule caps (diameter: 7.2 mm) as well as the flaps were lifted prior to chemical fixation (formalin) to expose the lamellar beds. The infrared femtosecond laser used in this study was the FS 200 platform (WaveLight GmbH, Erlangen, Germany). It emitted at a wavelength of 1030 nm and was operated with a pulse energy of 800 nJ, a spot separation of 7 mm x 7 mm, a pulse duration of ~ 300 fs, and a repetition rate of 200 kHz (total fluence: 1.6 J/cm^2^). Again, these parameters reflect the common clinical settings. The FS 200 was employed for flap creation only (same flap parameters as with the ultraviolet laser).

### Sample preparation and data acquisition

Immediately after every attempt to create a flap or refractive lenticule, AS-OCT (TOMEY SS-100, Tomey Inc. Nagoya, Japan) was used to identify the cutting lines by the bright appearance of the cavitation gas produced intraoperatively^16,19^. Discernible cutting lines in the AS-OCT images were counted as an indicator for successful flap or lenticule creation. Absence of the bright cutting lines was interpreted as a strong indicator for cutting failure. Thereafter, manual extraction of the lenticules or lifting of the flaps was attempted. In case of successful lenticule extraction, a side cut was generated and the lenticule cap was lifted like a flap. Subsequently, the eyes were further processed for SEM, as described previously^15,19,20^. In brief, the globes were immersion-fixed for 24 h at room temperature in a solution containing 10% formalin and 2.5% glutaraldehyde in phosphate-buffered saline (PBS) [pH 7.2]. After several rinses in PBS, the corneas were dissected from the globes and trimmed for the lasered area. This was ensued by post-fixation in 1% osmium tetroxide and dehydration in an ascending series of alcohols and acetone. Thereafter, the samples underwent critical point drying using the Leica EM CPD300 system (Leica Mikrosysteme GmbH, Vienna, Austria), whereupon they were mounted on aluminium stubs (Ted Pella Inc., Redding, CA, USA) with conductive silver (Plano GmbH, Wetzlar, Germany). Then, the specimens were sputter coated with gold (15 nm) applying the Leica EM ACE200 system (Leica Mikrosysteme GmbH, Vienna, Austria). Qualitative morphological examination of the corneal samples was performed using the JSM-IT 300LV scanning electron microscope (JEOL Germany GmbH, Eching, Germany).

## Results

### Human eyes

**Fig. 2:**
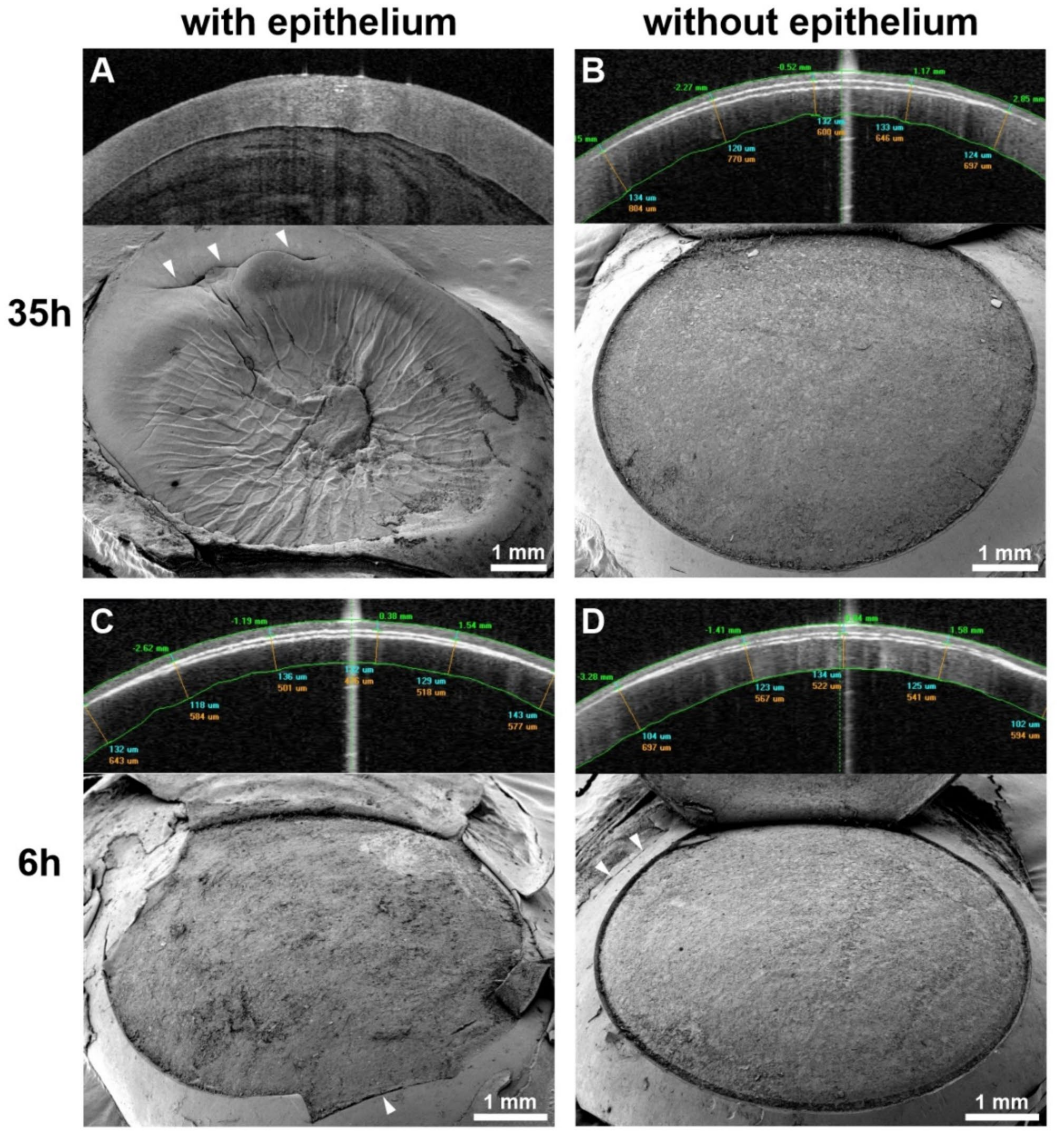
Human corneas after attempted stromal lenticule creation with a UV femtosecond laser. The upper portion of each partial figure shows a cross-sectional AS-OCT image of the cornea, the lower portion displays a scanning electron micrograph of the stromal lenticule beds. (**A**) Unsuccessful attempt at 35h p.m. without abrasion of the corneal epithelium. No cutting lines visible in the AS-OCT image. The SEM image illustrates that the corneal stroma is not exposed due to the incapacity of the UV laser to cut beneath the corneal epithelium. The crater-like appearance of the corneal surface is a technical artifact due to insufficient intraocular pressure during fixation. (**B**) Successful attempt at 35h p.m. after abrasion of the corneal epithelium. The stromal UV laser cuts present themselves as white cutting lines along the anterior and posterior lenticule interfaces in the AS-OCT image. SEM shows an oblique view on the exposed lamellar bed after lenticule extraction and lifting of the lenticule cap. (**C**) Successful attempt at 6h p.m. without abrasion of the corneal epithelium. Cutting lines and lenticule bed are depictable via AS-OCT and SEM, respectively. (**D**) Successful attempt at 6h p.m. after abrasion of the corneal epithelium. Cutting lines and lenticule bed are depictable via AS-OCT and SEM, respectively. Arrowheads: Corneal incision at the superficial end of an extraction canal.

**Fig. 3:**
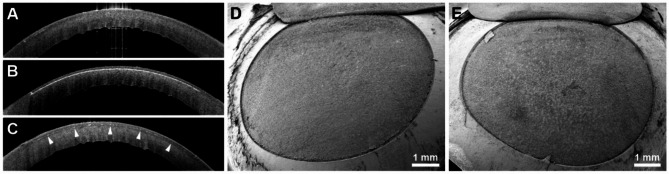
Human corneas after flap creation at 33h p.m. with ultraviolet and infrared femtosecond lasers. (**A**–**C**) AS-OCT images of corneal cross-sections. (**D**,** E**) SEM images of lamellar beds after successful flap lift (oblique views). (**A**) Unsuccessful attempt by UV laser without prior abrasion of the corneal epithelium. No cutting line discernible. (**B**) Successful attempt by the UV laser in the same specimen after abrasion of the corneal epithelium. White cutting line along the interface discernible in the anterior stroma. (**C**) Successful attempt by an infrared femtosecond laser in the contralateral eye without abrasion of the corneal epithelium. The white cutting line appears thin but discernible (arrowheads). (**D**) Stromal bed of a flap created by the UV laser after abrasion of the corneal epithelium. The interface appears smooth and regular. (**E**) Stromal bed of a flap created by an infrared laser without prior abrasion of the corneal epithelium. The interface appears smooth and regular.

Of the 6 attempts of UV femtosecond laser treatment in 6 different human eyes with the corneal epithelium still in place, only one had been successful. Only the eye with a postmortem time of 6 h showed clear signs of UV femtosecond laser cuts despite the corneal epithelium being still intact. Here, refractive lenticule extraction and cap lift were possible. The cutting lines were easily discernible in the corresponding AS-OCT image (Fig. 2C, upper part) and the lenticule bed surface was accessible after lifting and reflection of the lenticule cap. The interface exhibited pronounced surface roughness and conspicuous irregularities (Fig. 2C, lower part). In all other corneas that still possessed their epithelium and that had been subjected to considerably longer postmortem times (see Table 1), the creation of stromal cutting planes was impossible with the UV femtosecond laser. Consequently, no cutting lines were discernible in AS-OCT (Figs. 2A, 3A) and the corneal stroma remained inaccessible by SEM (Fig. 2A) in these cases. After abrasion of the corneal epithelium, stromal lenticule and flap creation worked well with the UV laser, irrespective of the postmortem time. Thus, the corresponding AS-OCT images displayed clear cutting lines (Figs. 2B,D and 3B) and SEM analysis yielded smooth and regular lenticule beds (Figs. 2B,D and 3D). The infrared femtosecond laser was able to create a morphologically normal stromal flap through an intact corneal epithelium after 33 h postmortem (Fig. 3C,E).

### Porcine eyes

**Fig. 4:**
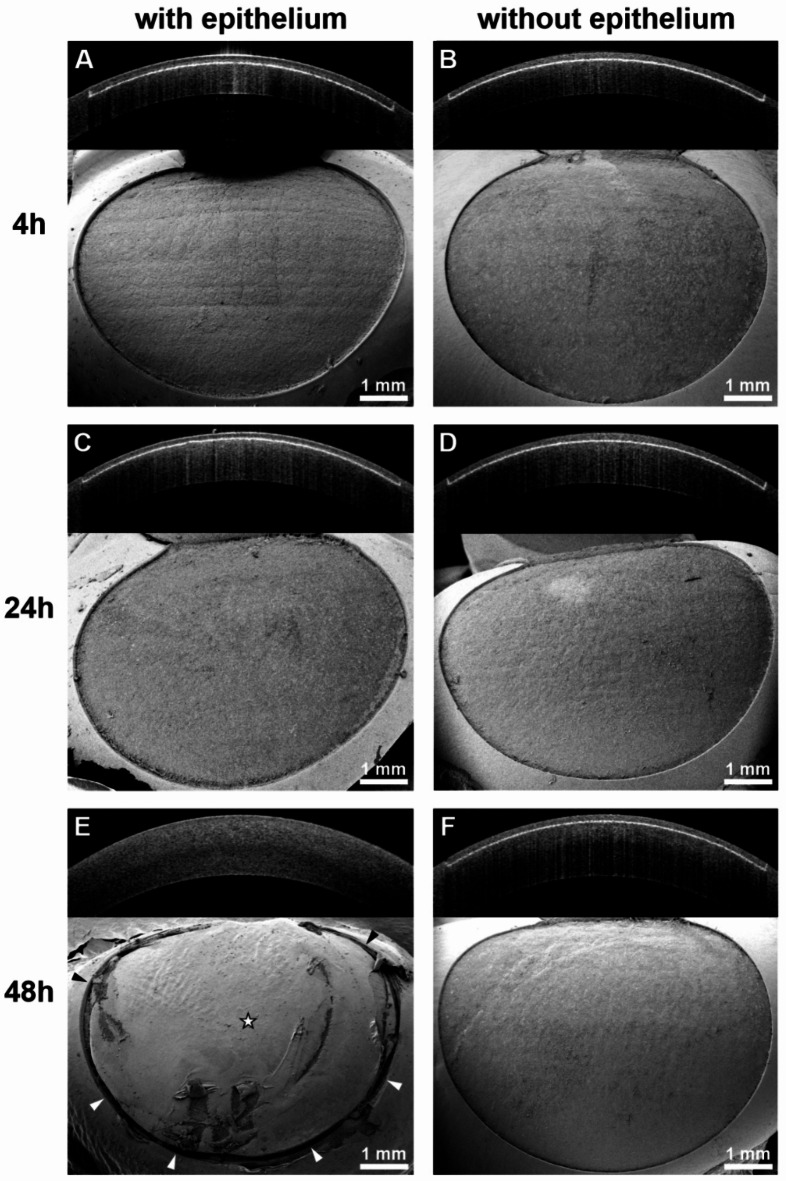
Porcine corneas after attempted stromal flap creation by the UV femtosecond laser. The upper portion of each partial figure shows a cross-sectional AS-OCT image of the cornea, the lower portion displays a scanning electron micrograph of the stromal flap beds. In all successful attempts, the white cutting lines were easily discernible in the AS-OCT images and the exposed flap beds were demonstrable after flap lift via SEM. (**A**) Successful attempt at 4h p.m. without prior abrasion of the corneal epithelium. (**B**) Successful attempt at 4h p.m. after abrasion of the corneal epithelium. (**C**) Successful attempt at 24h p.m. without prior abrasion of the corneal epithelium. (**D**) Successful attempt at 24h p.m. after abrasion of the corneal epithelium. (**E**) Unsuccessful attempt at 48h p.m. without prior abrasion of the corneal epithelium. The flap outline (asterisk) is demarcated by a superficial corneal incision (arrowheads). However, insertion of a blunt spatula and dissection of the flap remained impossible. Conspicuous scratches in the corneal epithelium of the intended flap are indicative of several failed dissection attempts. (**F**) Successful attempt at 48h p.m. after abrasion of the corneal epithelium.

**Fig. 5:**
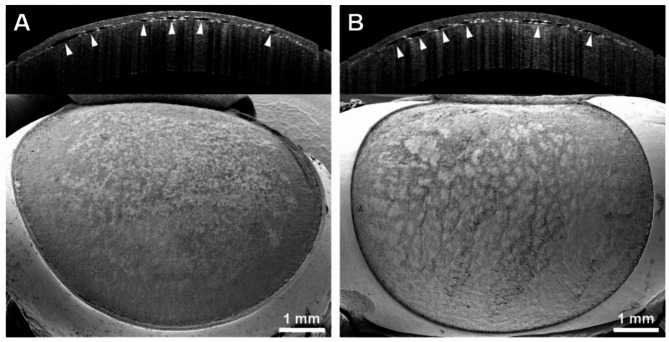
Porcine corneas after stromal flap creation at 48h p.m. by an infrared femtosecond laser. The upper portion of each partial figure shows a cross-sectional AS-OCT image of the cornea, the lower portion displays a scanning electron micrograph of the stromal flap beds. (**A**) Without prior abrasion of the corneal epithelium. (**B**) After abrasion of the corneal epithelium. Flap creation was feasible with the infrared laser in both groups without any difficulties or marked differences. Irrespective of the presence or absence of the corneal epithelium, the cutting lines were discernible in the AS-OCT images and the stromal flap beds appeared smooth and regular in SEM. Arrowheads: gas bubbles at the interface along the cutting lines.

After abrasion of the corneal epithelium, stromal flap creation was successful with the UV femtosecond laser, irrespective of the postmortem times. Consequently, clear cutting lines and smooth lamellar beds were observed with AS-OCT and SEM, respectively, in all 30 eyes devoid of the corneal epithelium. This was true for the 4 h p.m. subgroup (*n* = 10; Fig. 4B), as well as for the 24 h p.m. (*n* = 10; Fig. 4D) and 48 h p.m. (*n* = 10; Fig. 4F) subgroups. The outcome was the same in all corneas with the epithelium still in place after 4 h p.m. (*n* = 10; Fig. 4A) and 24 h p.m. (*n* = 10; Fig. 4C). However, after 48 h p.m., flap creation was impossible with the UV laser in all 10 samples possessing an intact corneal epithelium. Thus, the interfaces were neither found with a blunt spatula, nor by use of AS-OCT (Fig. 4E). Since no flap lift was possible in these cases, the lamellar bed was not accessible for surface analysis. Therefore, the corresponding SEM images only show the outer surface of the still closed stromal flaps (Fig. 4E). Infrared femtosecond laser-assisted flap creation was successful in all 20 porcine eyes treated after 48 h p.m., irrespective of the presence or absence of the corneal epithelium (Fig. 5). Thus, the lamellar beds were discernible by AS-OCT and observable via SEM in both groups after 48 h postmortem. In all 20 porcine eyes subjected to infrared femtosecond laser-assisted flap creation, the morphological quality of the lamellar beds was satisfactory in terms of their regularity and surface smoothness. The presence of the corneal epithelium had no detectable adverse effect on the application of the infrared femtosecond laser after 48 h postmortem (Fig. 5).

## Discussion

Refractive femtosecond lasers are capable of cutting in the depth of the corneal stroma via the principle of laser-induced optical breakdown (LIOB)^21^. This means that the laser focus ionizes the stromal tissue and generates a plasma initiating a cavitation bubble^11,22,23^. The rapidly expanding plasma creates a shock wave that exerts considerable photomechanical stress on the cornea^24,25^. This is partly intended, because it further disrupts the stromal tissue and contributes to the cutting process. However, this laser-induced “micro-explosion” also brings about collateral damage that must be kept in check while still allowing the laser to create a cutting line/plane^26–28^. One of the solutions to this problem lies in the controlled application of ultrashort laser pulses at an energy level that is just about high enough to safely create a stromal cut. The use of ultraviolet instead of infrared laser light allows for a substantial reduction in the minimum pulse energy necessary for LIOB^12,14^. Due to their shorter wavelength, ultraviolet femtosecond lasers are capable of creating a markedly smaller focus than infrared femtosecond lasers^12^. Therefore, the pulse energy will be concentrated in a markedly smaller volume, which is the reason why the threshold for LIOB is decreased. Thus, ultraviolet femtosecond laser systems are able to cut more precisely and with less photomechanical stress, when compared to conventional infrared platforms. However, the latest high-end low-energy infrared laser systems like the FEMTO LDV Z8 (Ziemer Ophthalmic Systems AG) are also capable of cutting with pulse energies below 100 nJ due to small laser spots applied by lenses with a high numerical aperture at a repetition rate above 5000 kHz^29,30^. The use of ultraviolet light brings about photochemical hazards of its own^31,32^ that had to be carefully investigated to exclude additional risks for the patients seeking refractive treatment. Preclinical studies based on porcine end rabbit eyes had already shown that the ultraviolet femtosecond laser system used here is safe and effective enough to enter the clinical testing phase^16–18^. The first clinical results look promising since the UV laser seems to work very well in human patient eyes and the attempted KLEx procedures had been successful (unpublished data). Therefore, the problems encountered while applying the ultraviolet femtosecond laser on postmortem human donor eyes came as a surprise. Since these problems disappeared after abrasion of the corneal epithelium, it was concluded that postmortal changes of the corneal epithelial cells must be responsible. From the successful UV laser-enabled lenticule extraction after 6 h p.m. without prior abrasion of the corneal epithelium it was further deduced that the adverse postmortal effects need time to develop and that the underlying processes may progress with time. Essentially, these two hypotheses were verified in porcine eyes, albeit with some noticeable deviations regarding the timelines. In the human system, 15 h p.m. were already too late for the ultraviolet femtosecond laser to create stromal cuts without prior epithelial abrasion. Even after 6 h p.m., the extraction of the lenticule had not been easy, which resulted in a rough and irregular surface morphology of the lenticule bed. In porcine corneas, on the other hand, UV laser-assisted stromal flap creation worked perfectly fine even after 24 h p.m. without prior abrasion of the corneal epithelium. Only in the 48-hour group did the presence of the corneal epithelium make flap creation by the UV laser impossible. This discrepancy between human and porcine eyes in terms of their postmortal timelines is a clear limitation of the present study. An explanation for this finding is at present speculative and requires further investigation. There are several possible factors that may have contributed to these differences. Firstly, porcine eyes were taken from organisms at an average age of approximately 0.5 years, whereas the age-range of the body donors was 69–88 years in the present study. It is therefore tempting to speculate that the overall viability of the corneal epithelium and its resistance to postmortal alterations was more pronounced in the porcine eyes used in the present study than in human donor eyes. Secondly, the porcine eyes were harvested immediately after the kill. Hence, the corneal epithelium had not been covered by the eyelids postmortally. In human donors, eye enucleation takes place several hours (sometimes > 24 h) after death. This means that the corneal epithelium had been exposed to a very specific microenvironment under the cover of the eyelids for a considerable amount of time. Since this happened in a lifeless organism, the situation must have differed profoundly from the conditions during sleep. The lack of blood supply and tear production may have substantially and unphysiologically altered the biochemical environment of the corneal epithelium. Due to its direct contact with the (dead or dying) conjunctiva, aberrant reactions between the corneal epithelial cells on one side, and the aqueous, mucous, and fatty secretions of the lacrimal gland, the conjunctiva, and the Meibomian glands as well as other glands within the eyelids that contribute to the tear film on the other side, are conceivable. This setting could also favor hypoxic insult and the growth of certain microorganisms. Thirdly, the porcine eyes were kept refrigerated almost immediately after enucleation. Human body donors, on the other hand, are usually kept at room temperature for several hours after death, before being transferred to a cooling chamber. This is likely to accelerate or alter certain postmortal bioreactions^33^. Lastly, there is the additional probability of differences between porcine and human corneal epithelial structure and physiology, which may also account for – or at least contribute to – the disparity found. If and how exactly the described factors translate into the species-specific differences observed in the present study, remains unknown and is up for future research. The same is true for the distinct postmortal processes and mechanisms within the corneal epithelium that cause the deteriorating feasibility of UV femtosecond laser-assisted refractive surgery. The meticulous investigation of these confounding factors for UV laser-assisted refractive surgery may be of importance clinically, to rule out adverse effects of certain pathologies or medications – especially since abrasion of the corneal epithelium is not an option for patients seeking refractive correction via lenticule extraction.

## Conclusions

Based on the findings in human donor eyes, it was hypothesized that….

1. …postmortal changes in the corneal epithelium impair the ability of refractive UV femtosecond lasers to create stromal cuts.
2. …these changes develop and progress with time in the postmortal interval.
3. …these changes do not affect infrared femtosecond laser systems.

All three hypotheses were verified using porcine eyes, despite certain species-specific differences in the temporal development of the adverse epithelial changes. Although the corneal epithelium is markedly thicker in pigs than in humans^34–36^, the time until manifestation of these detrimental effects was demonstrated to be substantially longer in the porcine system. It is advisable to dedicate future research to the ascertainment of the postmortal cellular mechanisms responsible for the described limitation of ultraviolet femtosecond laser applicability. However, it is important to emphasize that none of these problems has been encountered clinically in patient eyes. Therefore, this seems to be solely an issue concerning the use of ex vivo eyes in a laboratory setting.

## Data Availability

The datasets generated during and/or analyzed during the current study are available from the corresponding author on reasonable request.
